# The Site of St. Mark's Hospital

**Published:** 1918-05-04

**Authors:** Chas. A. Hanson

**Affiliations:** Lord Mayor, President.


*To the Editor of* The Hospital.

### The Site of St. Mark's Hospital.

*To the Editor of* The Hospital.

Sir,—I venture to appeal to you to assist me in preventing what I fear will be a calamity to one of our City charities.

St. Mark's Hospital for Cancer, Fistula, and other Diseases of the Rectum, of which I am the president, needs the sum of £3,000 to complete the purchase at £5,500 of an adjoining site, on which otherwise a factory will be erected. The erection of such a building, with no restriction as to height or light, would not only be injurious to the hospital, but, what is much more serious, it would interfere adversely with the recovery of the patients, all of whom have undergone operations, many of them of a most serious nature.

The value of the special treatment at this hospital has been recognised by the War Office, which has arranged to take over a number of the beds for the special treatment of soldiers wounded in the bowel. Many have already passed through the hospital, and the efforts of the surgical staff have been crowned with success. If, however, this factory is allowed to be erected, the recovery of these poor wounded soldiers will be greatly impeded through the absence of sufficient light and air.

The vendors have granted the first option of purchase to the hospital, conditionally upon the purchase money being paid by June 30. Over £2,500 has already been raised, and several other donations have been promised if the balance can be secured. Will you assist me with a donation, however small, and so save this charity from the grave situation with which it is threatened?—Yours faithfully.

Chas. A. Hanson,

April 1918. Lord Mayor, President.

[We have already drawn attention to the facts described in the Lord Mayor's widely circulated letter of appeal, and if the additional £3,000 required is to be raised by June 30 no time must be lost.—Ed. The Hospital.]

